# Cohabitation, Relationship Stability, Relationship Adjustment, and Children’s Mental Health Over 10 Years

**DOI:** 10.3389/fpsyg.2021.746306

**Published:** 2022-02-02

**Authors:** Heather M. Foran, Janina Mueller, Wolfgang Schulz, Kurt Hahlweg

**Affiliations:** ^1^Department of Health Psychology, University of Klagenfurt, Klagenfurt, Austria; ^2^Department of Clinical Psychology, Psychotherapy, and Assessment, Technical University of Braunschweig, Braunschweig, Germany

**Keywords:** marriage, cohabitation, child externalizing symptoms, relationship adjustment, adolescence

## Abstract

Understanding risk factors for relationship dissolution and poor relationship adjustment among couples has been an active area of research in relationship science. One risk factor, non-marital cohabitation, has shown to predict higher rates of relationship dissolution and relationship instability in some samples, but the associations among German parents with children over time are less clear. In this study, we examined the links between non-marital cohabitation and 10-year outcomes (relationship dissolution, relationship adjustment over time, and child internalizing and externalizing symptoms) in 220 German families with preschool-aged children at the initial assessment followed into adolescence. Families were assessed 7 times over the 10 years and retention at the 10-year follow-up was over 92%. After accounting for multiple testing, only mother’s report of better initial interparental communication predicted whether parents were likely to stay together over the course of the 10 years. Adolescents of parents who cohabited were at higher risk for externalizing symptoms 10 years later compared to children of married parents. In addition, although there were no differences between cohabiting couples and married couples at the initial assessment in relationship adjustment, cohabiting mothers who stayed with their partner over the 10 years showed significantly greater declines in relationship adjustment over the 10 years compared to married mothers. Findings indicate the need for further research that explores family structure and dynamics over time to inform refinement of prevention programs targeting relationships and children’s mental health.

## Introduction

In recent years, cohabitation without marriage has become a more socially accepted family structure in many westernized countries (Cunningham and Thornton, 2005; Sassler and Lichter, 2020). Approximately 50% of women reported cohabiting with a partner as a first union, with 40% of these transitioning to marriage within 3 years, 27% ending the relationship, and 32% remaining in a cohabiting relationship (Copen et al., 2013). Likewise, there has been an increase in the number of families with children who are cohabiting in many countries over the last half century (Bumpass and Lu, 2000; Kreider, 2005; Kennedy and Bumpass, 2008). Approximately half of children under 16 in the United States are estimated to live with a mother in a cohabiting relationship at some point during their childhood (Kennedy and Bumpass, 2008).

Similar to the United States, Germany has also experienced increasing rates of cohabitation and non-marital births (Perelli-Harris et al., 2018). According to the most recent statistics, the number of cohabiting couples in Germany has almost doubled to 843,000 since 1996 (BMFSFJ, 2017). The non-marital birth rate has also risen significantly. In 2015, 35% of all new-born children were born to parents who were not married, compared to 10% in 1950 (BMFSFJ, 2017). Of relevance, German social policies and taxation law continue to favor marriage over cohabitation and provides incentives for marital childbearing (e.g., financial advantages, tax splitting, spouse insurance, parental rights in the case of joint legal custody) (Schnor, 2014; Perelli-Harris et al., 2018).

The choice to cohabitate rather than marry may reflect views about the institution of marriage and its importance, economic reasons, or other selection differences between those who choose to cohabitate or marry (Kline et al., 2004; Stanley et al., 2004, 2006). Past research with samples from the United States has found that cohabiting couples often differ from married couples. Couples who cohabit rather than marry have lower education (McGinnis, 2003), are more equalitarian in gender roles (Le Bourdais and Lapierre-Adamcyk, 2004), and come from more unstable family backgrounds (Kamp Dush et al., 2003). In some countries, economic barriers to marriage may be more pronounced among couples with children who cohabit (Lichter, 2012).

Findings regarding the differences between non-marital cohabitating and married couples in relation to child and relationship outcomes has been mixed (Amato, 2015; Sassler and Lichter, 2020). Cohabitating relationships are less stable than married relationships in many countries (Italy, Great Britian, and Scandinavia: Thomson et al., 2019; Germany: Bastin et al., 2012; Sweden: Kennedy and Thomson, 2010; United States: Kennedy and Bumpass, 2008; Australia: Wilkins et al., 2010). In some studies, cohabitating couples are also at risk for lower commitment to the relationship (Stanley et al., 2004) and more depressive symptoms (Stafford et al., 2004; Kamp Dush, 2013). However, accounting for demographic and other contextual factors, differences may not hold and not all studies find significant differences (Amato, 2015; Sassler and Lichter, 2020).

Given the mixed findings in the literature, it is important to better understand whether cohabitation also predicts relationship dissolution and dissatisfaction among couples with children and to examine whether this is linked with the mental health of children. There is limited long-term research on this topic with samples of German parents but results of cross-sectional and longitudinal studies in the United States support non-marital cohabitation among parents as a risk factor for some poor outcomes among children (e.g., Brown, 2004; Artis, 2007). In one Norwegian study of women followed over the transition to parenthood, women who cohabited reported less relationship satisfaction over the 18-month follow-up period compared to married women (Mortensen et al., 2012). In a study of the United Kingdom Millennium Cohort, the risk for separation before the child’s fifth birthday was 26% for cohabiting parents compared to 9% for married parents (Callan et al., 2006). Further, cohabitation (as well as single status) is associated with increased risk of poor birth health outcomes compared to children of married mothers (Shah et al., 2011). Thus, non-marital cohabitation was linked to an increase in family instability and also to negative implications for children’s health outcomes (Amato, 2001; Osborne and McLanahan, 2007; Kalil et al., 2011; Kim, 2011). Recent research also found that children born to or raised by cohabiting parents are more likely to exhibit internalizing and externalizing problems, show more aggressive behaviors, and experience more difficulties with social relationships than do children born to married parents (Amato, 2001; Brown, 2004; Fomby and Osborne, 2010; Goldberg and Carlson, 2014).

One explanation for these findings may not be relationship status but rather due to the quality of the interparental relationship. Children are particularly at risk when parents’ relationships are conflictual and discordant (Amato et al., 1995; Rhoades, 2008). Higher parental conflict is associated with higher behavioral problems and maladjustment among children (Amato, 2001; Osborne and McLanahan, 2007; Kalil et al., 2011; Kim, 2011; Goldberg and Carlson, 2014; Davies et al., 2016). Further, longitudinal data suggest that interparental conflict is associated with decreases in positive parenting and children’s emotional security, which in turn predicts the development of externalizing and internalizing behavior problems in children (Schacht et al., 2009).

Another explanation for cohabitation being linked with children’s health and parent’s relationship outcomes may be through societal mechanisms. In most European countries non-marital cohabitation has developed into a socially accepted alternative for individuals in close relationships. Compared to the United States, where non-marital cohabitation is often viewed as a stepping stone to marriage (Sassler and Lichter, 2020), cohabitation has become a common form of partnership in Germany, especially for younger birth cohorts (Nazio and Blossfeld, 2003). In fact, data from the German youngest birth cohorts suggested that about 40% of women in Eastern Germany and about 50% in Western Germany have adopted cohabitation before eventually entering into first marriage (Nazio and Blossfeld, 2003). Further, studies found that German couples have even more positive views of living in a cohabitation relationship without marriage intentions than couples in Great Britain and Australia (Treas et al., 2014; Perelli-Harris et al., 2019). This suggests that despite German family policy benefits to marriage, cohabitation may be more tolerated in the German society and less stigmatized (Perelli-Harris et al., 2019). As social stigma or social norms against premarital cohabitation has worn off in Germany, one might expect the effect on child mental health and relationship outcomes through social stigma or social norms may be less applicable in comparison to other countries in which non-marital family structures are more stigmatized.

In the present study, we examine whether parents with preschool-aged children (ages 2.5–6 years old) who cohabit or are married are at differential risk for relationship dissolution over the span of 10 years using a prospective sample of German families. As most of the research has been conducted on United States samples rather than international samples, the focus on German parents fills a gap in the literature (Jose et al., 2010). German parents are particularly interesting because, in Germany, there is relatively low levels of social disapproval against non-marital cohabitation compared to other countries (Lappegård et al., 2014). There is also little research that has examined these associations past early childhood into adolescence (Bulanda and Manning, 2008). It is unclear whether parental non-marital cohabitation will relate to adolescent mental health, but it possible that as adolescents start to form their own dating relationships associations with their parent’s relationship history may be significant.

In particular, we were interested in addressing three research questions. First, we were interested in determining whether initial relationship status (non-marital cohabitation vs. marriage) predicted whether couples separated over a 10 year follow-up period **(R1)**. Parents who cohabit or are married may differ on other variables (e.g., sociodemographic factors or initial relationship quality, initial relationship communication), which may account for any differences in dissolution rates observed. To consider this possibility, we were interested in testing whether differences in rates of relationship dissolution were retained after accounting for any other identifiable differences between cohabiting parents and married parents at the initial assessment.

Second, we were interested in whether cohabiting or married parents who remained together over the 10-year period differed in how satisfied they were over time **(R2)**. We hypothesized that cohabitation at the initial assessment would predict steeper declines in relationship adjustment over the 10 year period based on findings from other studies followed over shorter periods of time (e.g., Mortensen et al., 2012).

Our third research question was to test whether parental intimate relationship variables predicted the presence of significant externalizing and internalizing symptoms among the children, now adolescents, at the 10-year follow-up after controlling for initial symptoms during preschool ages **(R3)**. We examined whether initial relationship adjustment, initial relationship communication, initial relationship status, and relationship dissolution over time predicted adolescent externalizing and internalizing symptoms as reported by mothers on the widely used Child Behavioral Checklist (Achenbach and Rescorla, 2000).

## Materials and Methods

### Participants and Procedure

Participants were recruited from preschools in Braunschweig, Germany (see Heinrichs et al., 2005 for more details on the recruitment process) to participate in a randomized controlled trial of a universal primary parenting prevention program (i.e., the Triple-P positive parenting program; Sanders, 1999). Briefly, 17 preschools were selected in order to yield a sample representative of a range of social-economic status using the social index of their catchment area *via* the objective Kita Social Index. Parents, fluent in German, were eligible to participate if they had a child 2 1/2–6 years old attending preschool. Preschools were used for recruitment of a representative sample since most children in Germany attend preschool (“Kindergarten”) due to their widespread availability and low cost. The population response rate was 31% (*N* = 280) of those invited to participate (Heinrichs et al., 2005), similar to other international prevention trials (Sanders, 1999). Only parents who were cohabiting or married at pre-assessment were eligible for the current study (*N* = 220).

Participants were assessed 7 times over the course of the 10-year study (baseline, approximately 6 months following the initial assessment, 4 additional times every 12 months after the pre-assessment, and 10 years later). Participant retention was excellent across the 10 years; 92.3% of families provided data over the 10 year time period (*n* = 203 of the initial 220 cohabiting or married parents). Participants were given 50 Euros for participating in the first assessment. They were provided 20 Euros for all subsequent assessments. This study was approved by the university IRB board and informed consent was provided.

The mean age of the sample was 38.8 (6.0) years old for men and 35.6 (4.5) years old for women at baseline. The target child was 4.0 years old on average at baseline (*SD* = 0.97). The majority of the sample reported having an income in the middle range (55%, 1,500–3,000 Euros per month after taxes); 37% reported income greater than 3,000 Euros per month; 5% of the sample reported income of less than 1,500 Euros per month and 3% did not report income information. Eighty-eight percent of men and 9% of women reported working full-time; 2% of men and 47% of women reported working part-time; and 44% of women and 9% of men were unemployed.

In Germany, there are three levels of secondary education (high, middle, and low). Over half of men and women (63 and 58%, respectively) had completed the high level (typically indicative of individuals who attend college); 22% of men and 34% of women completed the middle level (typically indicative of individuals who obtain some specialized training other than a bachelor’s degree) and 16% of men and 7% of women reported the low level (typically indicative of individuals who do not complete high school). Regarding post-secondary education, half of the men (53%) and 37% of women had completed some type of university degree; 12% of men and 27% of women had completed a specialized training or community college degree and 36% of men and women had completed an apprenticeship or had no post-secondary education. The number of children living in the household was 2.1 (*SD* = 0.84) on average.

### Measures

#### Relationship/Marital Status

Parents’ initial relationship status was measured at baseline assessment among both members of the couple with a categorical variable (married vs. cohabitating, with the higher value indicating cohabitation).

#### Relationship Stability (Staying Together)

Separation was assessed at the 10-year follow-up with categorical value the categories of “partnered” and “divorced or separated at any time” in the 10-year period; the higher value indicated staying with the same partner over the 10-year period.

#### Relationship Adjustment

The 7 item Abbreviated Dyadic Adjustment Scale (ADAS; Sharpley and Rogers, 1984; Köppe, 2001) was used to assess relationship satisfaction (e.g., “How often do you and your partner have a stimulating exchange of ideas.”) over the course of the 10 years (7 time points). Items are scored on a Likert scale from 0 to 5, with higher scores indicating more relationship satisfaction (mothers at baseline α = 0.81, fathers at baseline α = 0.82).

#### Couple Problem-Solving Communication

Couple communication over the 10 years was assessed with the 7 item communication scale (Christensen and Sullaway, 1984; Kröger et al., 2000) rated on a 9 point scale ranging from 1 to 9. Participants were required to indicate the likelihood to which both partners contribute to a discussion and try to solve problems when an issue or problem arises (e.g., “both spouses express feelings to each other,”; “both spouses blame, accuse, or criticize each other”). Internal consistency with this measure was high across both genders (mothers at baseline α = 0.89, fathers at baseline α = 0.88). This measure assesses interparental communication, including conflictual communication and was assessed at baseline in the current paper.

#### Child Internalizing and Externalizing Symptoms

The commonly used Child Behavior Checklist was used to assess mother-reported child internalizing and externalizing symptoms at the initial assessment and at the 10-year follow-up (Achenbach and Rescorla, 2000). This widely used measure asks parents to report the presence and frequency of child behavioral problems (e.g., hits others) and emotional problems (e.g., rapid changes between sadness and excitement) using a three step format (0 = not true; 1 = somewhat or sometimes true; 2 = very true or often true). The internal consistencies are high in this sample (internalizing symptoms at baseline α = 0.87, externalizing symptoms at baseline α = 0.90). The age-appropriate German versions of the Child Behavior Checklist (CBCL 1 1/2–5 and CBCL 4–18) for children aged 1 1/2–5 years and 4–18 years were used at pre-assessment and the CBCL 4–18 was used at the 10-year follow-up. Since the two age-dependent versions cannot be directly compared, scores were converted to Z scores at the pre-assessment (in accordance with the recommendation from the author; T. Achenbach, personal communication, March 2008) and analyzed as a continuous variable due to the version differences. Ten year outcomes were the presence of internalizing symptoms and externalizing symptoms at or above the borderline to clinically significant cut-offs. Approximately 20% of children had externalizing problems and 23% had internalizing symptoms at the 10-year follow-up.

### Analytical Strategy

For all analyses, models were run using full information maximum likelihood estimation with robust statistics in Mplus (Muthén and Muthén, 2012). Prior to testing the first research question (**R1**), we examined any baseline differences between cohabitors and married parents using *t*-tests or chi-square tests, where appropriate. Any significant differences were included as covariates in the models predicting whether couples stayed together at the 10-year follow-up. The covariate examined included whether they participated in the Triple P parenting program, whether they participated in the 10-year follow-up or dropped out, child age, parental age, child gender, number of siblings, child behavioral or emotional problems, parenting skills, couple communication, relationship adjustment, parental depressive symptoms, parental anxious symptoms, parental secondary education, post-secondary education, family income and stress levels. Two regression models were tested for mothers and fathers. The first model included initial relationship status and relationship communication at the pre-assessment, and controlled for any pre-assessment differences between cohabiting and married parents. The second model included relationship satisfaction instead of communication due to their shared variance (i.e., collinearity).

For the second research question (**R2**), we sought to examine whether relationship status predicted relationship adjustment over time among parents who stayed together. We used latent growth curve analyses to examine the trajectories of relationship adjustment over the 10-year period. Models were run separately for men and women to examine the impact of relationship status on the relationship adjustment of each gender (rather than the couple) and due to the smaller sample size for fathers. Specifically, we examined whether the latent slope of relationship adjustment over the 10 years was predicted by relationship status at pre-assessment.

In the last set of analyses for research question three (**R3**), we conducted regression analyses in a structural equation modeling framework with MPlus statistical software to test whether relationship status at the initial assessment, relationship stability over the course of the 10 years, and initial relationship adjustment predicted the presence of mother reported children’s internalizing and externalizing symptoms at the 10-year follow-up. A variety of the initial assessment variables were included in the model as controls including demographic variables, treatment condition, and mental health symptoms of children. None of the covariates besides baseline mental health symptoms of the children were significant, so only this variable was retained in the models. Analyses were run a second time included relationship communication at the initial assessment as a predictor rather than relationship adjustment at the initial assessment.

## Results

### Relationship Status and Separation Over Time (R1)

Descriptive statistics and correlations for study variables are provided in Supplementary Material for fathers and mothers. Fifty percent of cohabiting parents and 17.1% of married couples at baseline separated over the 10 year period based on analyses with participants who reported data at the 10 year follow-up (*n* = 203). Rates were similar when the full sample was analyzed regardless of dropout time point (52.2% of cohabitating parents vs. 15.7% of married parents, based on sample *N* = 220) or when only parents who provided data at least through the 4 year follow-up were analyzed (52.2% of cohabitating parents vs. 16.2% of married parents, based on sample *n* = 214).

To consider that pre-assessment differences between cohabiting and married parents may explain a difference in rates of separation, we compared cohabiting and married parents at pre-assessment on study variables with independent *t*-tests or chi-square tests. There were no significant differences at pre-assessment between cohabiting parents and married parents on whether they participated in the Triple P parenting program, whether they participated in the 10 year follow-up or dropped out, child age, child gender, number of siblings, child behavioral or emotional problems, parenting skills, couple communication, relationship adjustment, parental depressive symptoms, parental anxious symptoms, parental secondary education, mother’s post-secondary education, or mother’s reported stress levels (*p*s > 0.05). There were, however, significant differences at pre-assessment based on a few sociodemographic variables such that cohabiting parents were younger (mothers *t* = 3.61, *p* = 0.000, fathers *t* = 3.04, *p* = 0.003) and reported less monthly family income than married parents (*t* = 4.69, *p* = 0.000). In addition, cohabiting fathers reported more stress (*t* = –2.49, *p* = 0.014) and less post-secondary education than married fathers (fathers χ^2^ = 8.05, *p* = 0.018).

Results for RQ1 are shown in Table 1. Models for mothers included the covariates age and family income. Due to the covariance between relationship adjustment and communication (*r* = 0.71), models were estimated with each of these variables separately. Results in Table 1 show that relationship communication at pre-assessment significantly predicted relationship stability at 10 years after accounting for all significant pre-differences. For men, there were more covariates included in the model since more differences based on relationship status were observed at baseline. Income, not shown, was also tested and did not significantly predict relationship status in any of the models. This variable was removed since it reduced the sample size due to some missing data on income at baseline, which reduces sample size even when using procedures such as full information maximum likelihood to account for missingness. There were no significant predictors in the men’s models after accounting for multiple testing with a Bonferroni correction.

**TABLE 1:** Baseline predictors of relationship stability (staying together) over the 10 years.

	β	S.E.	*t*	*p*
**Women’s models**				
Maternal age	0.08	0.08	0.99	0.332
Family income	0.03	0.07	0.42	0.677
Communication	0.19	0.07	2.75	0.006*
Cohabitation	–0.22	0.10	–2.28	0.023
Maternal age	0.08	0.08	1.00	0.317
Family income	0.05	0.07	0.67	0.503
Relationship adjustment	0.12	0.08	1.59	0.113
Cohabitation	–0.20	0.10	–2.09	0.037
**Men’s models**				
Paternal age	–0.07	0.10	–0.75	0.453
Education	0.06	0.07	0.79	0.429
Stress	0.02	0.09	0.20	0.843
Communication	0.06	0.08	0.79	0.430
Cohabitation	–0.20	0.10	–1.97	0.049
Paternal age	–0.06	0.10	–0.63	0.527
Education	0.06	0.08	0.74	0.457
Stress	0.02	0.09	0.26	0.795
Relationship adjustment	0.08	0.09	0.99	0.322
Cohabitation	–0.19	0.10	–1.96	0.050

n = 194–201 mothers; n = 186 fathers. Father models: Income and education were correlated (r = 0.49). The sample size was reduced when income was included (n = 179). Income was not a significant predictor in any of the models so results are also presented with it excluded. Cohabitation = A negative coefficient indicates that those who cohabit are less likely to stay together. Bonferroni correction applied, *p < 0.013.

### Relationship Status and Relationship Adjustment Over Time in Long-Term Relationships (R2)

To further examine the course of relationship adjustment over time for cohabiting couples and married couples, the means and standard deviations of relationship adjustment over time for couples who stayed together and were married or cohabitating are presented in Table 2 (*n* = 161) for each year time point. Using latent growth curve analyses, latent slope of relationship adjustment over the 10-year follow-up was predicted from relationship status (cohabiting or married at pre-assessment). When including covariates as described above and listed in Table 1 (e.g., age and income for women), the pattern of results did not change. The model was estimated with maximum likelihood estimation with robust statistics to account for non-normality in the data using Mplus 7.1 statistical software (Muthén and Muthén, 2012). The growth curve slope for relationship adjustment was modeled based on time of the assessment (0, 0.5, 1, 2, 3, 4, and 10). The linear model was a good fit to the data for mothers (χ^2^ = 67.95, df = 33, CFI = 0.95, TLI = 0.95) and fathers (χ^2^ = 43.44, df = 33, CFI = 0.98, TLI = 0.98). Relationship status predicted a declining slope for relationship adjustment over the 10-year follow-up for mothers (*b* = –0.35, SE = 0.17, *Z* = –2.10, *p* = 0.036), but not for fathers (*b* = 0.12, SE = 0.14, *Z* = –0.89, *p* = 0.375). Thus, cohabiting mothers exhibited a 0.35 point greater decrease (or smaller increase) in relationship adjustment per year than did married mothers. To further explore these trends, we examined mean differences in relationship adjustment over time. There was no significant difference in mean relationship adjustment among cohabiting and married parents at earlier time points, but at the 10-year time point, cohabiting mothers who had remained with their partners, reported lower levels of relationship adjustment than married mothers who remained in their relationships (*p* = 0.011).

**TABLE 2:** Relationship adjustment of those couples who stayed together over the 10 Years.

	Pre	1 year	2 years	3 years	4 years	10 years
**Married women**						
Mean	23.19	23.81	23.27	23.64	23.81	20.30
SD	4.86	4.74	4.97	4.92	5.27	5.65
**Cohabiting women**						
Mean	22.00	21.91	22.18	22.82	21.33	15.73
SD	5.08	3.68	3.27	3.76	4.46	6.11
**Married men**						
Mean	23.60	23.67	24.05	24.27	23.82	23.76
SD	4.79	4.46	5.12	4.93	5.18	5.42
**Cohabiting men**						
Mean	22.03	22.93	22.34	23.18	23.39	22.09
SD	4.13	3.47	3.45	5.09	6.50	6.35

*n = 161 intact relationships over 10 years.*

### Baseline Relationship Variables, Relationship Stability and Children’s Internalizing and Externalizing Symptoms at the 10-Year Follow-Up (R3)

Lastly, we examined whether relationship variables significantly predicted mothers’ report of children’s externalizing and internalizing symptoms at the 10-year follow-up. Mothers’ report was used for these analyses due to the higher retention among mothers compared to fathers. Results are shown in Table 3. Cohabitation at pre-assessment significantly predicted more externalizing symptoms of children at the 10-year follow-up; externalizing symptoms of children at pre-assessment was also a significant predictor of 10-year outcome. For internalizing behaviors at 10-year follow-up, only pre-assessment internalizing behaviors significantly predicted 10-year report of internalizing behaviors.

**TABLE 3:** Predictors of adolescent internalizing and externalizing symptoms at the 10-year follow-up.

	β	S.E.	*t*	*p*
**Internalizing symptoms at 10 years**				
**Model 1 *r*^2^ = 0.12**				
Internalizing symptoms	0.34	0.08	4.15	0.000*
Stayed together	–0.01	0.06	–0.11	0.910
Communication	–0.00	0.01	–0.42	0.672
Cohabitation	0.30	0.26	1.18	0.240
**Model 2 *r*^2^ = 0.12**				
Internalizing symptoms	0.34	0.08	4.35	0.000*
Stayed together	–0.01	0.06	–0.16	0.874
Relationship adjustment	–0.01	0.01	–0.41	0.679
Cohabitation	0.29	0.26	1.13	0.257
**Externalizing symptoms at 10 years**				
**Model 3 *r*^2^ = 0.17**				
Externalizing symptoms	0.38	0.07	5.66	0.000*
Stayed together	–0.06	0.06	–0.98	0.033
Communication	0.00	0.01	0.19	0.852
Cohabitation	0.69	0.27	2.57	0.010*
**Model 4 *r*^2^ = 0.18**				
Externalizing symptoms	0.41	0.27	2.67	0.008*
Stayed together	–0.07	0.06	–1.19	0.233
Relationship adjustment	0.02	0.01	1.85	0.064
Cohabitation	0.71	0.27	2.67	0.008*

When the covariates age, income, and treatment status were included, none of the results changed. All independent variables indicate baseline levels except staying together which reflects whether the couple stayed together (vs. separated/divorced) over the 10 year period. Bonferroni correction applied, *p < 0.013.

Analyses were run a second time with relationship communication at the pre-assessment as an independent variable in the models (see Table 3) rather than relationship adjustment. Relationship communication at pre-assessment was not a significant predictor of children’s externalizing or internalizing symptoms at the 10-year follow-up (*p*s > 0.05).

## Discussion

Several interesting and important findings emerged from the current study of families. Using a prospective sample followed over 10 years with over 92% retention at the 10-year follow-up among mothers, we examined the impacts of initial relationship variables on parental relationship outcomes and child mental health symptoms 10 years later. Results showed that cohabiting non-married parents were almost three times more likely than married parents to end their relationship over the course of the 10-year period (50 vs. 17%). However, after accounting for covariates and multiple testing, this difference in risk for dissolution was no longer statistically significant. The only significant predictor of relationship dissolution was interparental relationship communication at baseline reported by mothers. Although, among the cohabiting parents who stayed together over the 10 year period, cohabitation (compared to marriage) predicted significant declines in mother’s relationship adjustment over time.

Further, cohabitation also was associated with children’s externalizing symptoms. Children whose parents were cohabiting at the initial assessment were more likely to experience higher levels of externalizing symptoms 10 years later even after controlling for initial symptoms. We did not, however, find an association of cohabitation and children’s internalizing symptoms. These findings are consistent with existing research which have shown that parents’ relationship quality and children’s externalizing problems are reciprocally related, but not children’s internalizing problems (Fomby and Cherlin, 2007; Osborne and McLanahan, 2007; Fomby and Estacion, 2011; Goldberg and Carlson, 2014). It is not surprising that cohabitation associations were observed only for children’s externalizing problems and may be related to measurement issues. Adolescents’ report of their own internalizing symptoms may be more reliable than their mothers’ report since they may not disclose their own emotional symptoms to their mother (Goldberg and Carlson, 2014).

The emotional security theory (Davies and Cummings, 1994) may advance an understanding of how non-marital cohabitation relates with children’s externalizing symptoms. In the emotional security theory, interparental conflict has been shown to play a key role in risk for children’s poor adjustment (Cummings et al., 2006). Our findings indicate that parents’ initial relationship status is related to children’s externalizing symptoms, regardless of parents’ initial relationship adjustment, relationship communication, and relationship dissolution over time. Theoretically, cohabitation may increase feelings of emotional insecurity in the parental relationship regardless of parents’ initial relationship adjustment and relationship communication or it may be that destructive communication moderates or mediates the association with child maladjustment. Thus, it could be that the risk for externalizing symptoms based on early cohabitation status of parents may be accounted for interparental conflict more specifically. These possibilities could be explored in future studies.

Taken together, these findings partially support the importance of cohabitation for understanding the longitudinal parental relationship and child behavior outcomes. It is also interesting to note that other relationship variables, such as initial relationship adjustment and relationship communication, did not predict outcomes at 10 years for children. Thus, the results indicate that cohabitation may be a useful factor that identifies parents in need of relationship education programs, even when their initial relationship adjustment does not indicate a risk. Relationship education programs have been shown to be effective in many countries including Germany (Hawkins et al., 2008; Hahlweg and Richter, 2010).

These results should not be interpreted to imply that cohabiting relationships confer no benefits. Compared to single individuals and dating relationships both cohabitation and marriage show benefits for mental health (Osborne and McLanahan, 2007; Rhoades et al., 2009; Amato, 2015). In a representative United States sample, entering a cohabiting relationship or a marital relationship was followed by improvements in mental health (e.g., lower suicide risk and depressive symptoms) among individuals followed through their twenties. Nonetheless, there appears to be some features of cohabitation without marriage that place parents at higher risk for dissatisfaction over time. In our study, this was demonstrated among parents with children from preschool age to adolescence. A next step would be to examine these patterns among older adolescents entering new dating relationships to see whether findings can be replicated and expanded on with more rigorous assessments of family dynamics and structure over time.

Although this study identifies cohabitation as a significant predictor of relationship and child outcomes, it does not address the reason for the associations with cohabitation. One perspective is that those who cohabit are different from those who marry and this explains these differences in outcomes (called the social *selection* perspective, James and Beattie, 2012). A second perspective is that there is something intrinsic to the cohabitation *experience* over time that explains differences in outcomes. Previous studies have found support for the second perspective in such that initial differences do not account for cohabitation and its association with relationship stability and quality (Kamp Dush et al., 2003; James and Beattie, 2012).

Previous research also indicates that the association between cohabitation and marital dissolution particularly affects those who cohabited before engagement but not after engagement or not at all until marriage (Rhoades et al., 2009). Recent research also found that in the first year of marriages, couples who had cohabited before marriage had lower rates of marital dissolution compared to couples who did not cohabit before marriage. This finding on marital stability disappeared over time; meaning that premarital cohabitation may have short-term benefits for couples, but long-term costs for marital stability still remain (Rosenfeld and Roesler, 2019). Further studies should take these findings into account, as well as assess serial relationships, when evaluating the association between cohabitation, children’s mental health and parents’ relationship outcomes.

### Strengths and Limitations

There are several strengths of this study worthy of mention. In particular, the use of the prospective design across such a long time period of 10 years and the excellent retention across time adds to the confidence in the study results. Further, as far as we are aware, this is one of the only studies that has examined the effects of cohabitation among German parents followed over such a long time period. Other strengths include the use of statistical controls for other confounding effects and the examination of trajectories of relationship adjustment over time with latent growth curve modeling.

Moreover, as noted in the meta-analysis of cohabitation by Jose et al. (2010), there has been a relative dearth of studies with international samples. In Germany, cohabitation and raising children is more culturally accepted than it is in the United States, and accordingly, there is less stigma associated with such arrangements and less pressure to marriage. Although subcultural differences exist within German families in which pressure to marry may vary and cohabitation may be more or less accepted, by conducting this study in a country in which overall tolerance of non-marital family structures is more accepted, the results support the theory that the cohabitation findings are not fully explained by societal pressure. In a large study of Norwegian mothers followed from pregnancy for 18 months, cohabitation was also a predictor of lower relationship adjustment and this remained constant over time (Mortensen et al., 2012). Thus, in countries in which social disapproval of cohabitation is relatively low (Lappegård et al., 2014), the link between marital status and relationship stability among parents still emerge (see also Rosenfeld and Roesler, 2019). Other studies have suggested that the effects of cohabitation in comparison to marriage may be larger in countries where cohabitation is uncommon, less socially accepted and where traditional gender roles are common (Soons and Kalmijn, 2009; Lee and Ono, 2012). Of course, further studies would be needed to systematically test mechanisms through which cohabitation impacts parental relationship outcomes and children’s externalizing symptoms over time.

There are also several limitations. The sample size was adequate but not extremely large and there were less cohabiting couples than married couples. Findings will need to be replicated with a larger sample. In addition, the sample consisted of parents who participated in an RCT of a brief group parenting intervention 10 years earlier, which is an important consideration for generalizability of the study findings. Participation in the study program was controlled for in all analyses and was not a statistically significant predictor of any outcomes examined in the current sample. Further, although prospective, the study cannot confirm causal relationships between variables and we were not able to determine when separation or divorce occurred over the 10 years. An additional limitation due to parent’s participation the study program is that parent’s initial relationship status was assessed among children in a specific age range (2 1/2–6 years). Thus, it cannot be ascertained whether parents were cohabiting or married at their child’s birth.

One limitation related to the measurement of interparental conflict was solely assessed by parent’s communication behavior. Since interparental conflict can take many forms, future studies could be improved by including different measures that account for several aspects of interparental conflict. Studies that examine co-parenting practices in the context of relationship status changes such as separations are also needed as cooperative co-parenting has been shown to relate to less externalizing and internalizing symptoms in children post-divorce (Lamela et al., 2015). Lastly, although efforts were made to recruit a sample representative of the region sampled, only a third of eligible parents contacted through preschools agreed to participate in this study. This participation rate is similar to rates found in the existing literature, but nonetheless, the current sample may differ from the population of parents in ways which are unknown.

### Clinical Implications

Non-marital cohabitation is increasingly common and should be more integrated into couple- and parenting-focused programs. Evidence-based relationship education programs may be especially useful in helping individuals at risk clarify their relationship’s future, in particular regarding to marital intentions and commitment (Rhoades et al., 2006). Further, an increasing number of studies indicate that relationship-focused programs for couples alone or combined with parenting programs are an effective way to strengthen marriage, parenting behavior, and improve children’s adjustment and behavioral development (Schulz et al., 2006; Zemp et al., 2006). Couple- and parenting-focused programs aimed at cohabiting non-married couples with or without children could address important factors such as family instability and change, mother’s relationship adjustment, children’s emotional security related to parent’s relationship status, the meaning of cohabitation, commitment levels, and other risk factors such as those related to destructive conflict communication (Kline et al., 2004; Rhoades et al., 2006; Stanley et al., 2006). Addressing such factors at an early stage might provide some buffer against any long-term negative effects on parental relationship outcomes and children’s behavioral problems and may foster emotional security in the family. Further studies are need that assess relationship changes dynamically among parenting samples and consider protective effects.

## Data Availability Statement

The raw data supporting the conclusions of this article will be made available by the authors, upon request and in compliance with data management procedures for this project.

## Ethics Statement

The studies involving human participants were reviewed and approved by the Technical University of Braunschweig IRB. Written informed consent to participate in this study was provided by the caregivers.

## Author Contributions

HF conceptualized the study, conducted the analyses, and wrote the first draft. JM contributed significant intellectual content, including some analyses and edits to revise the manuscript. WS and KH were involved in the original study on which this manuscript was based (Future Families Study) and contributed significantly to editing and revising the manuscript. All authors contributed to the article and approved the submitted version.

## Conflict of Interest

The authors declare that the research was conducted in the absence of any commercial or financial relationships that could be construed as a potential conflict of interest.

## Publisher’s Note

All claims expressed in this article are solely those of the authors and do not necessarily represent those of their affiliated organizations, or those of the publisher, the editors and the reviewers. Any product that may be evaluated in this article, or claim that may be made by its manufacturer, is not guaranteed or endorsed by the publisher.
