# Supplementary material for: Cohabitation, Relationship Stability, Relationship Adjustment, and Children’s Mental Health Over 10 Years
Source: Front Psychol. 2022 Feb 2;12:746306. doi: 10.3389/fpsyg.2021.746306 (PMC8847607; doi:10.3389/fpsyg.2021.746306)
Supplement: Supplementary file 1 [file Data_Sheet_1.PDF]

Supplementary Tables

Table A1

Women's Descriptive Statistics and Correlations

**Variables**	**1**	**2**	**3**	**4**	**5**	**6**	**7**	**8**	**9**	**10**	**11**	***M***	***SD***
1. Relationship Adjustment T1												22.8	5.1
2. Relationship Adjustment T2	.80**											23.5	4.9
3. Relationship Adjustment T3	.71**	.82**										23.4	5.0
4. Relationship Adjustment T4	.72**	.75**	.78**									23.0	5.1
5. Relationship Adjustment T5	.71**	.71**	.71**	.77**								23.3	5.3
6. Relationship Adjustment T6	.67**	.66**	.73**	.71**	.73**							23.5	5.7
7. Relationship Adjustment T7	.42**	.49**	.51**	.52**	.57**	.58**						20.5	5.8
8. Communication T1	.69**	.65**	.56**	.60**	.51**	.58**	.25**					5.7	10.4
9. Internalizing Symptoms T1	-.21**	-.15*	-.11	-.19**	-.21**	-.17*	-.10	-.32**				8.4	6.3
10. Internalizing Symptoms T7	-.14*	-.10	-.01	-.13	-.12	-.06	-.07	-.17*	.37**			6.4	5.9
11. Externalizing Symptoms T1	-.29**	-.22**	-.14*	-.21**	-.23**	-.24**	-.05	-.34**	.61**	.27**		11.6	7.4
12. Externalizing Symptoms T7	-.13	-.09	-.09	-.16*	-.11	-.12	-.08	-.23**	.30**	.47**	.47**	7.1	7.2

*$p < 0.05$, ** $p < 0.01$. Abbreviations: Relationship Adjustment (ADAS; Sharpley & Rogers, 1984; Köppe, 2001) scores range from 0 to 5 with higher scores indicating more relationship satisfaction. Communication (Kroeger et al., 2000; Christensen & Sullaway, 1984) scores range from 1 to 9 with higher scores indicating better couple communication. Higher child internalizing and externalizing symptoms (CBCL1 1/2–5 and CBCL 4–18) indicate more problems. T1 = baseline, T2 = 6 months, T3 = 1-year, T4 = 2-year, T5 = 3-year, T6 = 4-year and T6 = 10-year-follow-up.

Table A2

Men's Descriptive Statistics and Correlations

**Variables**	**1**	**2**	**3**	**4**	**5**	**6**	**7**	***M***	***SD***
1. Relationship Adjustment T1								23.3	5.0
2. Relationship Adjustment T2	.76**							23.2	4.6
3. Relationship Adjustment T3	.74**	.76**						23.5	4.7
4. Relationship Adjustment T4	.69**	.70**	.79**					23.5	5.1
5. Relationship Adjustment T5	.69**	.70**	.76**	.81**				23.8	5.3
6. Relationship Adjustment T6	.69**	.69**	.74**	.73**	.77**			23.4	5.6
7. Relationship Adjustment T7	.64**	.56**	.60**	.61**	.61**	.66**		23.8	5.8
8. Communication T1	.68**	.64**	.64**	.52**	.54**	.54**	.48**	5.2	10.5

*$p < 0.05$, ** $p < 0.01$. Abbreviations: Relationship Adjustment (ADAS; Sharpley & Rogers, 1984; Köppe, 2001) scores range from 0 to 5 with higher scores indicating more relationship satisfaction. Communication (Kroeger et al., 2000; Christensen & Sullaway, 1984) scores range from 1 to 9 with higher scores indicating better couple communication. Higher child internalizing and externalizing symptoms (CBCL1 1/2–5 and CBCL 4–18) indicate more problems. T1 = baseline, T2 = 6 months, T3 = 1-year, T4 = 2-year, T5 = 3-year, T6 = 4-year and T6 = 10-year-follow-up.
